# Association between early elevated phosphate and mortality among critically ill elderly patients: a retrospective cohort study

**DOI:** 10.1186/s12877-022-02920-z

**Published:** 2022-03-15

**Authors:** Jie Yang, Yisong Cheng, Ruoran Wang, Bo Wang

**Affiliations:** Department of Critical Care Medicine, West China Hospital, Sichuan University, No.37 Guo Xue Xiang St, Chengdu, 610041 Sichuan Province China

**Keywords:** Critical care, elderly patients, serum phosphate, independent risk factor, outcome

## Abstract

**Background:**

Phosphate disturbances are relatively common in hospitalized patients, especially in critically ill patients. The abnormal phosphate levels may indicate an abnormal body condition. However, little is known about the association between elevated serum phosphate and outcome in critically ill elderly patients. Therefore, the purpose of the present study was to investigate the association between early elevated phosphate and mortality in critically ill elderly patients.

**Methods:**

The present study was a retrospective cohort study based on the medical information mart for intensive care IV (MIMIC-IV) database. Patients with age ≥60 years old were enrolled in the present study. The primary outcome in the present study was ICU mortality. Univariate and multivariate Cox proportional hazard regression analyses were used to evaluate the association between early elevated phosphate and ICU mortality in critically ill elderly patients.

**Results:**

Twenty-four thousand two hundred eighty-nine patients were involved in this analysis and 2,417 patients died in ICU. The median age of involved patients was 78.4 (67.5, 82.9) years old. The median level of serum phosphate in the survivor group was 3.6 (3.0, 4.3) mg/dL, and the median level of serum phosphate in the non-survivor group was 4.4 (3.4, 5.8) mg/dL. The level of serum phosphate in the non-survivor group was significantly higher than the survivor group (4.4 vs. 3.6, *P*<0.001). The multivariate Cox proportional hazard regression demonstrated that elevated phosphate was an independent risk factor for ICU mortality, after adjustment for other covariates (HR=1.056, 95%CI: 1.028-1.085, *P*<0.001).

**Conclusions:**

In critically ill elderly patients, early elevated phosphate was significantly associated with increased ICU mortality.

## Background

Critically ill elderly patients are important components in critically ill patients in the intensive care unit (ICU). As an aging population, the number of critically ill elderly patients admitting to ICU is quickly increasing. The proportion of critically ill elderly patients was up to 20–30 percent of all admissions [1–3]. Why do we pay more attention to elderly patients? Because patients with old age tend to develop chronic illness and functional impairment, or generate poor outcomes [4–6]. Therefore, elderly patients, especially elderly critically patients require more concentration by physicians and careful treatments.

The development of electrolyte disorders is common in critically ill patients, such as hyponatremia, hypernatremia, hypomagnesemia, hyperkalemia, and the like [7]. These electrolyte disorders usually were reported to be associated with increased poor outcomes in critically ill patients, or be useful biomarkers for outcomes prediction [8–10]. Serum phosphate disturbances also belong to electrolyte disorders and develop frequently in hospitalized patients. Serum phosphate level elevates due to excretion by the kidney diminishes, especially in patients with kidney function injury [11–13]. Because critically ill patients develop organ failure including kidney injury frequently, serum phosphate disturbances are common in critically ill patients [14–16]. Thongprayoon and her (his) colleagues reported that admission hyperphosphatemia increased the risk of acute kidney injury in hospitalized patients [17]. George and her (his) colleagues found that hyperphosphatemia was also associated with an increased risk for mortality in severe burns [18]. Thus, Serum phosphate disturbances including hyperphosphatemia were a potential risk factor for poor outcomes in hospitalized patients.

Even though some related studies about serum phosphate were conducted and investigated that abnormal serum phosphate level was an independent risk factor for the development of poor outcomes in hospitalized patients, the relationship between serum phosphate on ICU admission and the mortality in critically ill elderly patients has not been explored and validated based on the big data. Therefore, the present study was designed and conducted based on the medical information mart for intensive care IV (MIMIC-IV) database to investigate the association between early serum phosphate on ICU admission and ICU mortality in critically ill patients.

## Material and methods

### Database source

Medical information mart for intensive care IV (MIMIC-IV, version 1.0) database is a single-center and big database containing real hospital stays for patients admitted to a tertiary academic medical center from 2008 to 2019 in Boston, MA, USA [19]. The MIMIC-IV database contains three modules including MIMIC-IV-Core, MIMIC-IV-Hosp, and MIMIC-IV-ICU. Patient demographics, clinical measurements, laboratory tests, treatments, pharmacotherapy, medical data, survival data, and more were included in the MIMIC-IV database. The researchers completing and passing the required training course could acquire access to this database. The consent for original data acquisition was obtained and the institutional review boards of the Massachusetts Institute of Technology and Beth Israel Deaconess Medical Center approved the establishment of the database. Therefore, patient informed consent and ethics approval were exempted for the present study. Data collected and presented in the present study were extracted by the author Yang and Cheng who completed and passed the required training course.

### Study population and data collection

The present retrospective cohort study was conducted based on the MIMIC-IV database. Patients were included in the present study if they were older than 60 years old and with available serum phosphate measurement records upon ICU admission within 24 hours. Patients were excluded if their length of ICU stay was less than 24 hours.

Patient medical data was extracted using PostgreSQL tools (version 13.0). The data including patient demographics and characteristics, signs and symptoms, laboratory findings, and treatment were collected when ICU admission within 24 hours. Of them, the maximum values of serum phosphate during the first 24 hours after ICU admission were included in the analysis. Patient outcomes were followed up in each ICU stay. The primary outcome in the present study was ICU mortality after 24 hours on ICU admission and the secondary outcomes were the length of ICU stay.

### Statistical analysis

Baseline characteristics and other clinical variables were compared between the survivors group and non-survivors group. Continuous variables were presented as mean ± standard deviation (SD) or median and interquartile range (IQR) according to their different distributions. A two-sample independent t-test was used for normal distribution or Mann–Whitney *U* test was used for non-normal distribution to compare differences between two groups. Categorical variables were presented as numbers and percentages, and compared by Chi-square test or Fisher’s exact probability test as appropriate. Univariate and multivariate COX proportional hazard regression analyses (variables presenting *P*<0.05 were included in multivariate regression analysis) were used to identify the association between early serum phosphate level and ICU mortality in critically ill elderly patients. The Lowess smoothing was also used to explore the curve relationship between serum phosphate level and ICU mortality. The receiver operator characteristic curve (ROC) and area under the curve (AUC) were used to evaluate the performance of early serum phosphate for prediction and calculate the cutoff value. The adjusted model using restricted cubic spline with 4 knots was conducted to flexibly represent the association between the hazard ratio and early serum phosphate as a continuous variable, using a reference level of the cutoff value of phosphate. The correlation between serum phosphate level and sequential organ failure assessment (SOFA) score was investigated using Spearman correlation analysis. *P*<0.05 was considered statistically significant in comparing differences between two groups, Cox proportional hazard regression models, and correlation analysis. All statistical analyses were performed by SPSS (version 22.0), STATA (version 16.0), and R software (version 4.0.4).

## Results

**Fig. 1:**
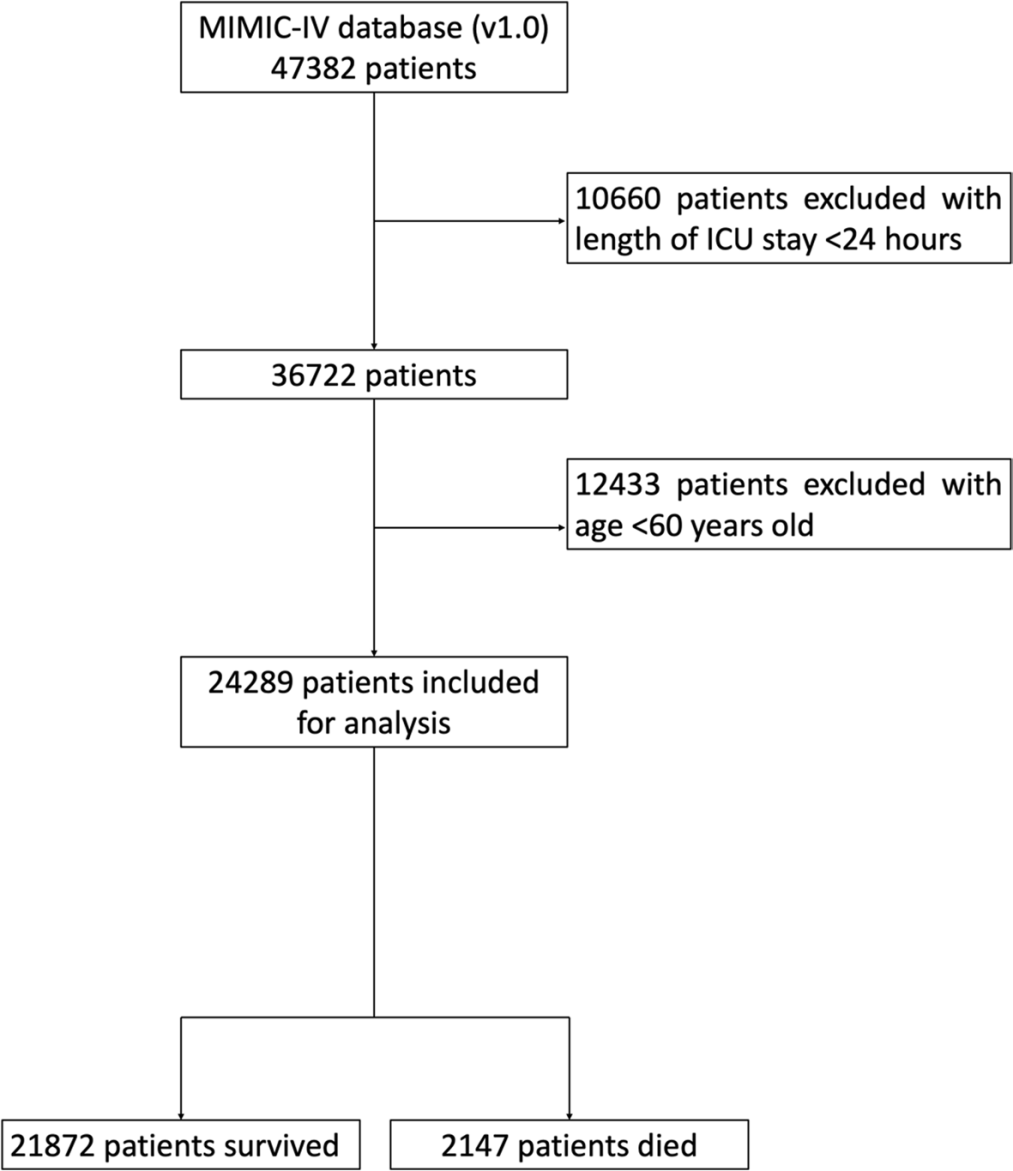
Study population

A total of 47,382 critically patients received an assessment. There were 24,289 critically ill elderly patients included in the present study and 2,147 patients died in ICU (Fig. 1). The median age of all patients was 74.8 years old and the non-survivors group was significantly older than the survivors group (76.7 vs. 74.5, *P*<0.001). The median SOFA score in the non-survivors group was significantly higher than the survivors group (11.0 vs. 5.0, *P*<0.001). As well as the SOFA score, the Charlson score indicating patient comorbidities was also higher in the non-survivors group (7.0 vs. 6.0, *P*<0.001). Other comparisons of demographics and clinical characteristics between the non-survivors group and survivors were also compared in Table 1.

**Table 1:** Comparisons between survivors and non-survivors

Variables	Total (*n* = 24289)	Survivors (*n* = 21872)	Non-survivors (*n* = 2417)	*P* value
Demographics and characteristics				
Age, year, median (IQR)	74.8 (67.5, 82.9)	74.5 (67.4, 82.3)	76.7 (68.7, 84.3)	<0.001
Male, no. (%)	13202 (54.4)	11913 (54.5)	1289 (53.3)	0.287
SOFA, median (IQR)	5.0 (3.0, 7.0)	5.0 (3.0, 7.0)	11.0 (7.0, 14.0)	<0.001
Charlson score, median (IQR)	6.0 (5.0, 8.0)	6.0 (5.0, 8.0)	7.0 (6.0, 9.0)	<0.001
Hypertension, no. (%)	11622 (47.8)	10643 (48.7)	979 (40.5)	<0.001
Diabetes, no. (%)	7970 (32.8)	7166 (32.8)	804 (33.3)	0.619
COPD, no. (%)	160 (0.7)	142 (0.6)	18 (0.7)	0.582
AKI, no. (%)	12505 (51.5%)	10688 (48.9%)	1817 (75.2%)	<0.001
Signs and symptoms				
Respiratory rate, median (IQR)	18.8 (16.7, 21.4)	18.6 (16.6, 21.1)	20.9 (18.1, 24.2)	<0.001
Heart rate, median (IQR)	81.7 (72.4, 92.8)	81.2 (72.0, 91.8)	88.6 (76.1, 101.9)	<0.001
Systolic pressure, mmHg, median (IQR)	116.5 (106.9, 129.0)	117.0 (107.5, 129.7)	110.0 (101.4, 122.1)	<0.001
Diastolic pressure, mmHg, median (IQR)	60.1 (54.1, 67.5)	60.3 (54.3, 67.7)	58.4 (51.2, 65.2)	<0.001
Mean arterial pressure, mmHg, median (IQR)	75.9 (70.0, 83.3)	76.2 (70.3, 83.6)	73.2 (67.4, 80.4)	<0.001
Temperature, °C, median (IQR)	36.8 (36.6, 37.0)	36.8 (36.6, 37.0)	36.8 (36.4, 37.1)	0.025
SpO_2_, %, median (IQR)	97.0 (95.6, 98.3)	97.0 (95.7, 98.3)	97.0 (95.1, 98.6)	0.351
Laboratory findings				
WBC count, ×10^9^/L, median (IQR)	10.8 (7.8, 14.5)	10.8 (7.8, 14.1)	12.5 (8.9, 17.9)	<0.001
Platelet count, ×10^9^/L, median (IQR)	192.0 (139.0, 250.0)	192.0 (140.0, 249.0)	192.0 (128.0, 258.0)	0.165
Hemoglobin, g/dL, median (IQR)	10.6 (9.0, 12.0)	10.6 (9.0, 12.0)	10.4 (8.8, 11.9)	0.007
Creatinine, mg/dL, median (IQR)	1.0 (0.8, 1.5)	1.0 (0.7, 1.4)	1.3 (0.9, 2.2)	<0.001
Urea, mg/dL, median (IQR)	21.0 (15.0, 33.0)	20.0 (15.0, 32.0)	30.0 (19.0, 49.0)	<0.001
Blood glucose, mg/dL, median (IQR)	131.5 (113.0, 159.3)	130.8 (112.7, 156.7)	144.4 (116.5, 185.0)	<0.001
Anion gap, mmol/L, median (IQR)	14.0 (12.0, 17.0)	14.0 (12.0, 16.0)	16.0 (14.0, 19.0)	<0.001
Sodium, mmol/L, median (IQR)	139.0 (136.0, 141.0)	139.0 (136.0, 141.0)	139.0 (135.0, 142.0)	0.176
Potassium, mmol/L, median (IQR)	4.1 (3.8, 4.5)	4.1 (3.8, 4.5)	4.3 (3.8, 4.8)	<0.001
Phosphate, mg/dL, median (IQR)	3.7 (3.1, 4.4)	3.6 (3.0, 4.3)	4.4 (3.4, 5.8)	<0.001
Urine within first 24h, mL, median (IQR)	3765.0 (2040.0, 7435.0)	3800.0 (2125.0, 7258.5)	3235.0 (881.0, 10020.0)	<0.001
Treatment				
Mechanical ventilation, no. (%)	11098 (45.7)	9162 (41.9)	1936 (80.1)	<0.001
Dialysis, no. (%)	937 (3.9)	690 (3.5%)	247 (10.2%)	<0.001
Other outcomes				
Length of ICU stay, hour, median (IQR)	59.0 (38.0, 107.0)	56.0 (37.0, 99.0)	102.0 (51.0, 205.0)	<0.001

*IQR *interquartile range, *SOFA *sequential organ failure assessment, *COPD *chronic obstructive pulmonary disease, *AKI *acute kidney injury, *SpO*_*2*_ pulse oximetry, *WBC *white blood cell, *ICU *intensive care unit

**Fig. 2:**
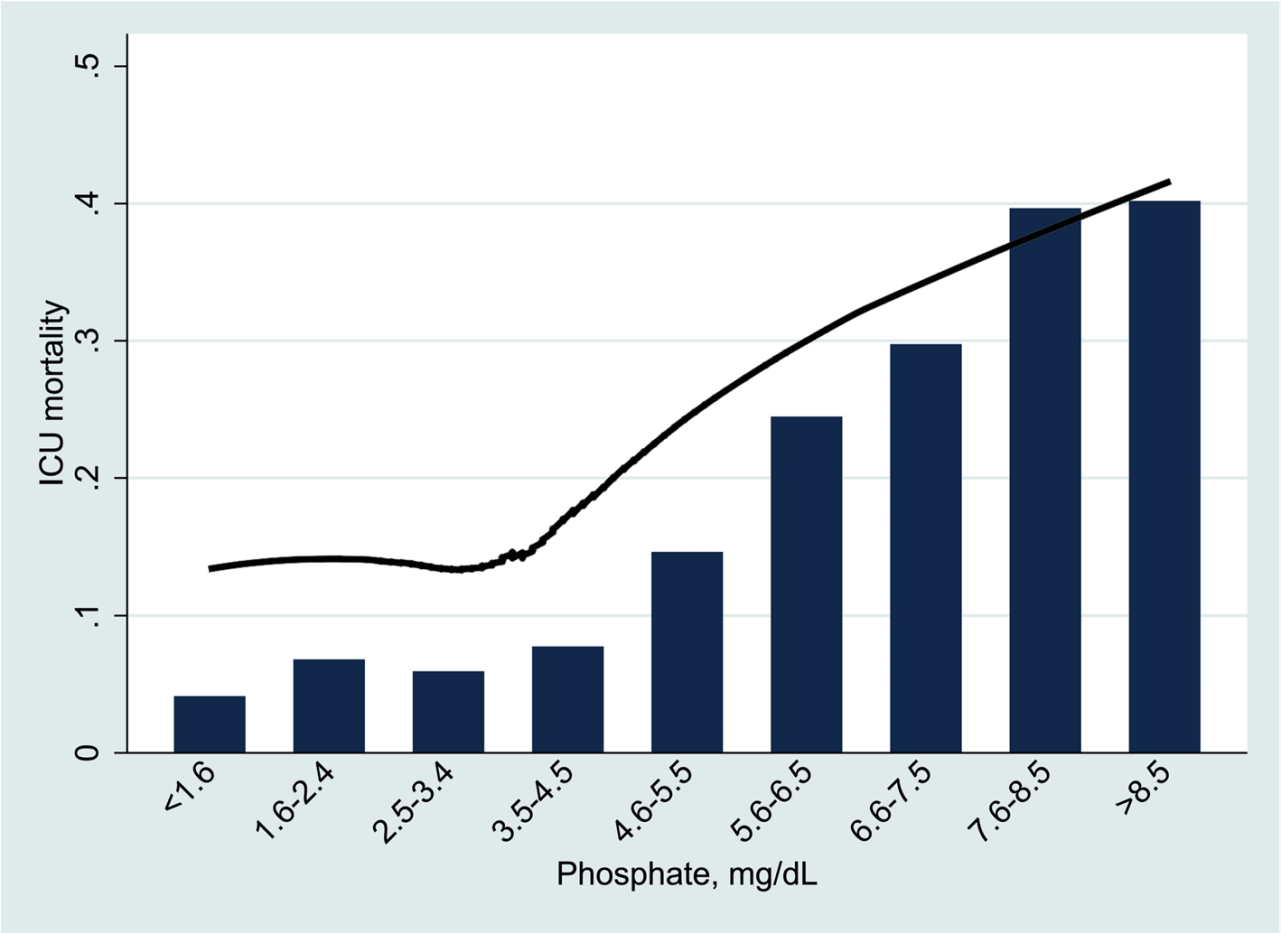
Association between early serum phosphate and the ICU mortality in critically ill elderly patients using Lowess smoothing

Table 2 demonstrated the results of univariate and multivariate Cox proportional hazard regression analyses. Early elevated serum phosphate was independently associated with increased ICU mortality in critically ill elderly patients (HR=1.056, 95%CI: 1.028-1.085, *P*<0.001) after adjustment other covariates and this result confirmed our hypothesis. Figure 2 using the Lowess smoothing also explored the curve relationship between early serum phosphate and ICU mortality. An elevated curve relationship between early serum phosphate and ICU mortality in critically ill elderly patients was found.

**Table 2:** Univariate and multivariate Cox proportional hazard regression analyses for primary outcome

	Univariate analysis		Multivariate analysis	
Variables	HR (95% CI)	*P* value	HR (95% CI)	*P* value
Age, year	1.024 (1.020-1.028)	<0.001	1.031 (1.026-1.036)	<0.001
SOFA	1.155 (1.145-1.165)	<0.001	1.122 (1.110-1.134)	<0.001
Charlson score	1.106 (1.090-1.123)	<0.001	1.054 (1.037-1.072)	<0.001
Respiratory rate	1.086 (1.076-1.096)	<0.001	1.045 (1.035-1.056)	<0.001
Heart rate	1.014 (1.012-1.016)	<0.001	1.005 (1.002-1.007)	<0.001
Mean arterial pressure, mmHg	0.973 (0.969-0.977)	<0.001	0.990 (0.986-0.995)	<0.001
SpO_2_, %	0.926 (0.912-0.939)	<0.001	0.964 (0.949-0.979)	<0.001
WBC count, ×10^9^/L	1.028 (1.023-1.033)	<0.001	1.009 (1.004-1.015)	<0.001
Hemoglobin, g/dL	0.971 (0.954-0.989)	<0.001	1.023 (1.004-1.043)	0.017
Urea, mg/dL, median (IQR)	1.010 (1.009-1.012)	<0.001	0.999 (0.998-1.001)	0.306
Blood glucose, mg/dL	1.000 (1.000-1.000)	0.463		
Anion gap, mmol/L	1.076 (1.068-1.083)	<0.001	1.026 (1.016-1.036)	<0.001
AKI	1.930 (1.759-2.118)	<0.001	1.152 (1.042-1.273)	0.006
Dialysis	1.768 (1.549-2.018)	<0.001	0.787 (0.681-0.911)	0.001
**Phosphate, mg/dL**	**1.213 (1.193-1.233)**	**<0.001**	**1.056 (1.028-1.085)**	**<0.001**

*HR *hazard ratio, *CI *confidence interval, *SOFA *sequential organ failure assessment, *SpO*_*2*_ pulse oximetry, *WBC *white blood cell, *AKI *acute kidney injury

**Fig. 3:**
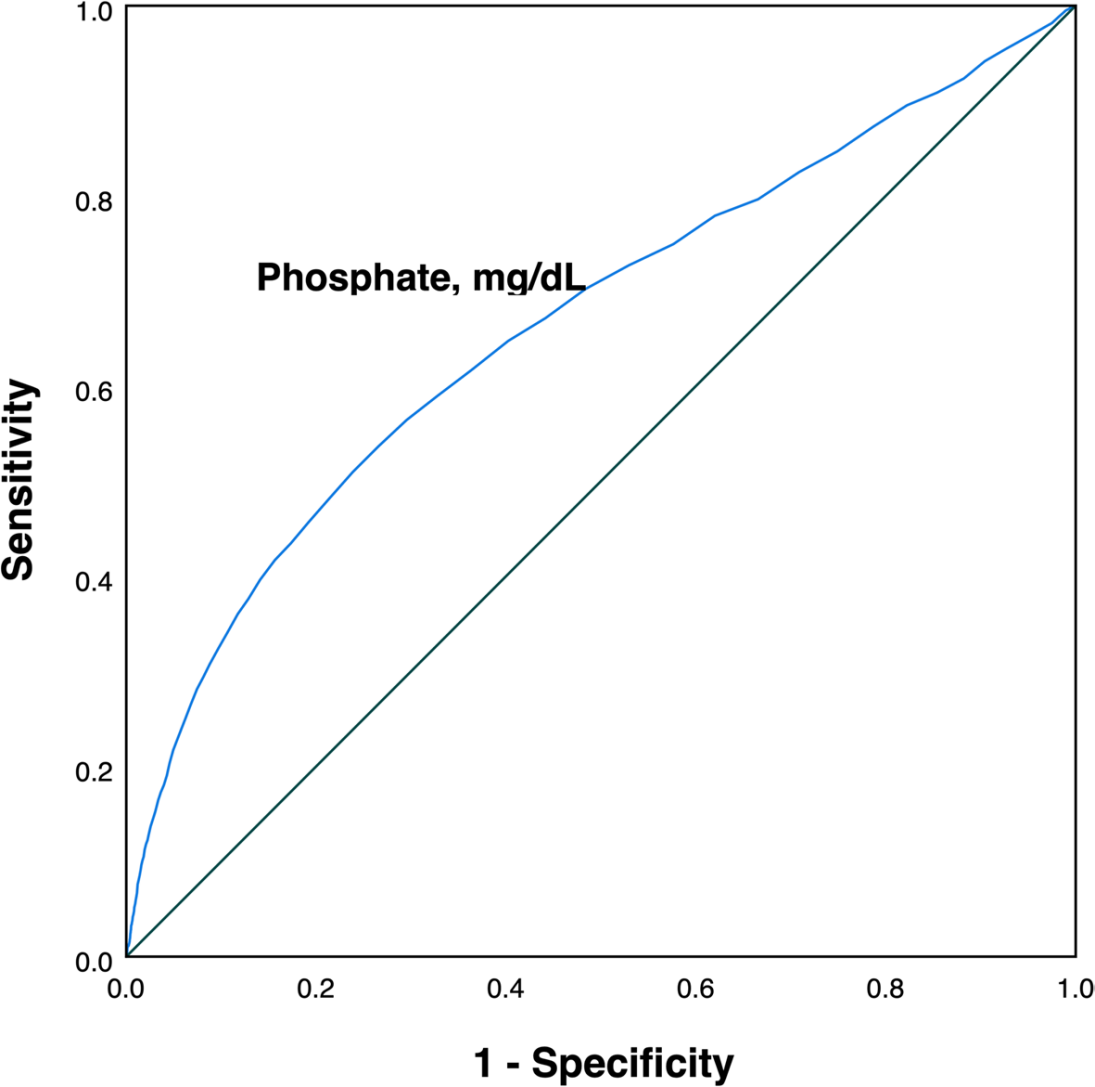
Receiver operating characteristic (ROC) curve analysis for early serum phosphate predicting ICU mortality in critically ill elderly patients. The AUC of early serum phosphate was 0.67

**Fig. 4:**
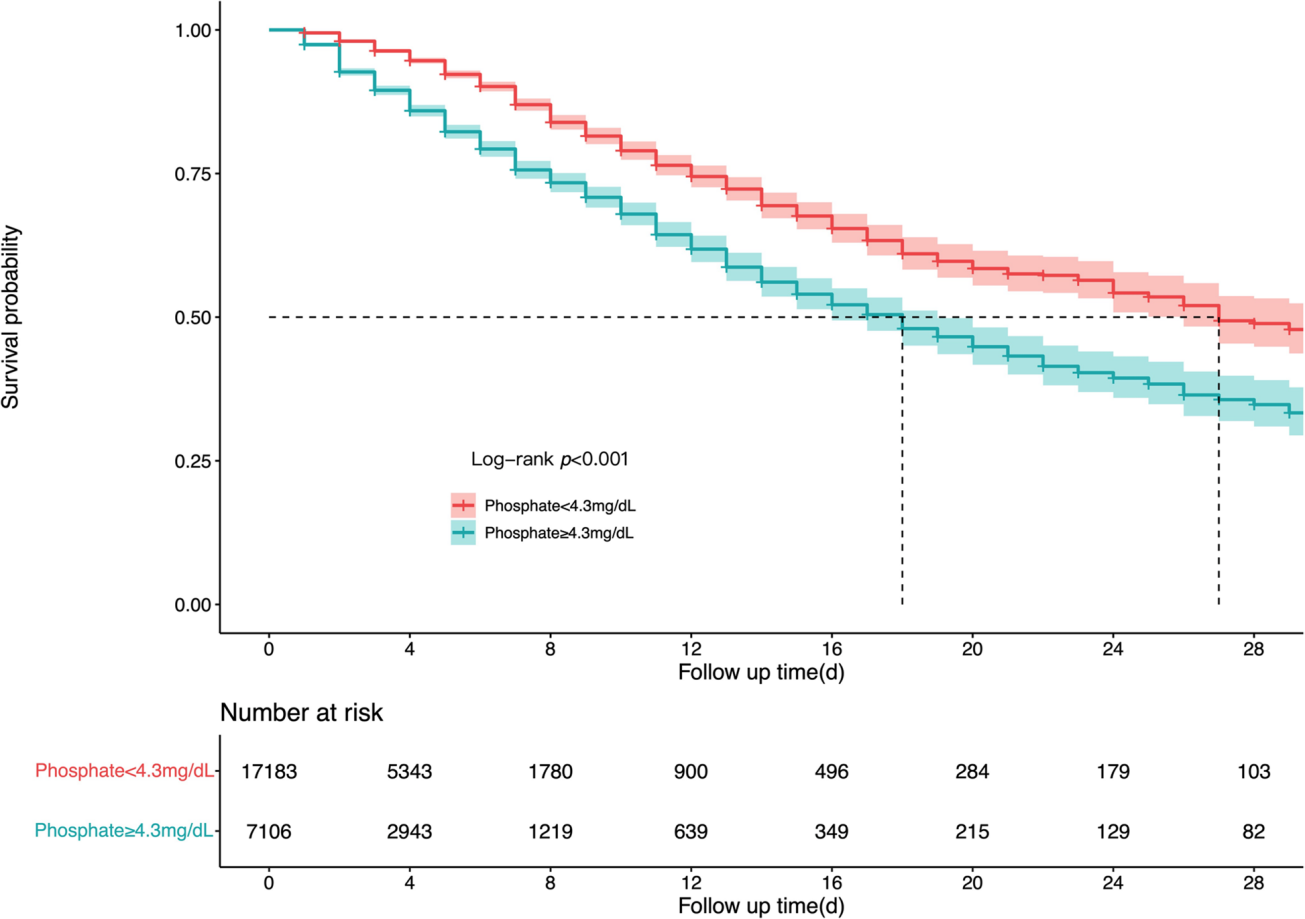
Kalpan-Meir survival curves of critically ill elderly patients with serum phosphate ≥4.3mg/dL and serum phosphate <4.3mg/dL

**Fig. 5:**
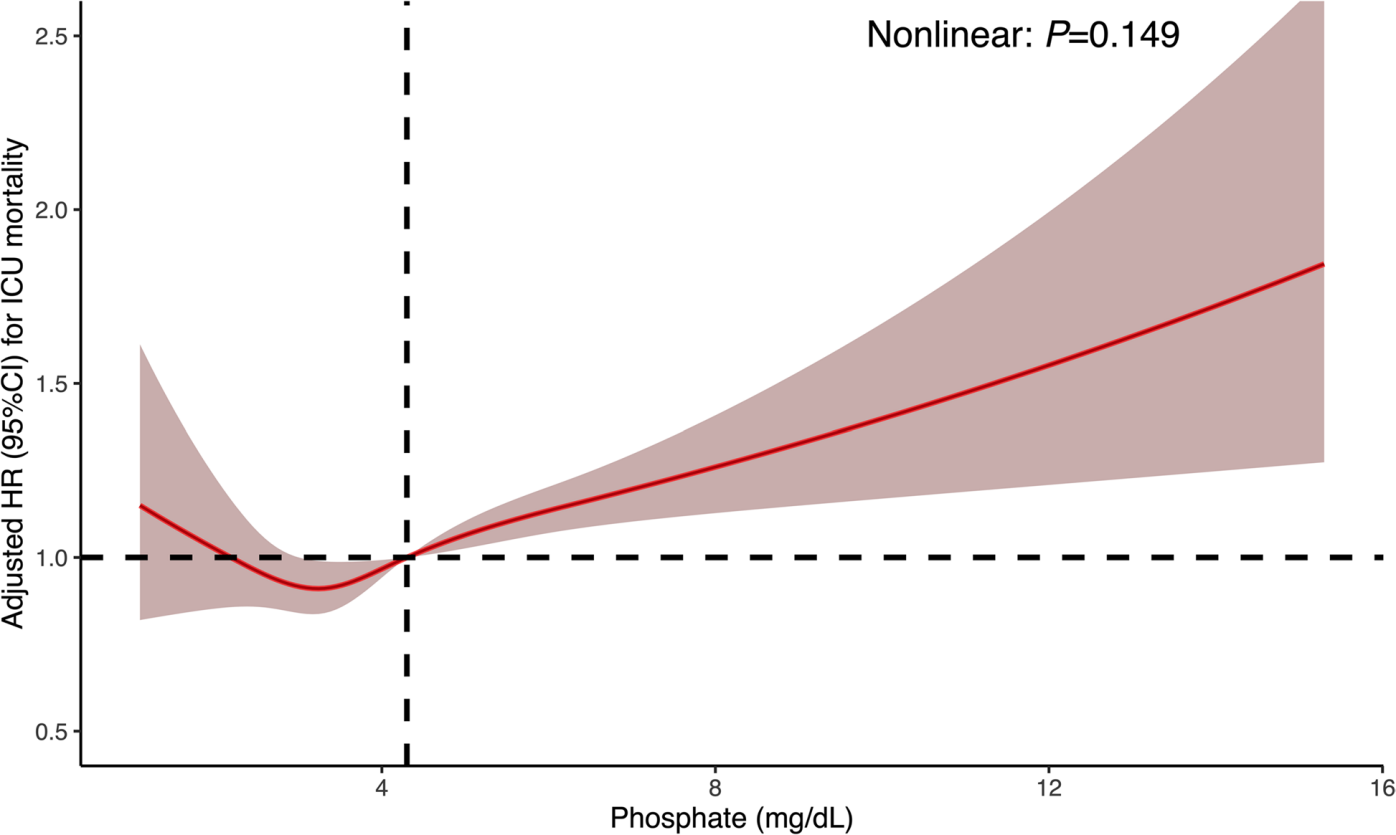
Association between early serum phosphate as a continuous variable and risk of ICU mortality of critically ill elderly patients. The analysis used a restricted cubic spline model with adjustment for age, SOFA score, Charlson index, respiratory rate, heart rate, mean arterial pressure, SpO_2_, WBC count, hemoglobin, urea, anion gap, AKI, and dialysis treatment. The reference (hazard ratio = 1, horizontal dotted line) was a serum phosphate of 4.3 mg/dL (vertical dotted line)

**Fig. 6:**
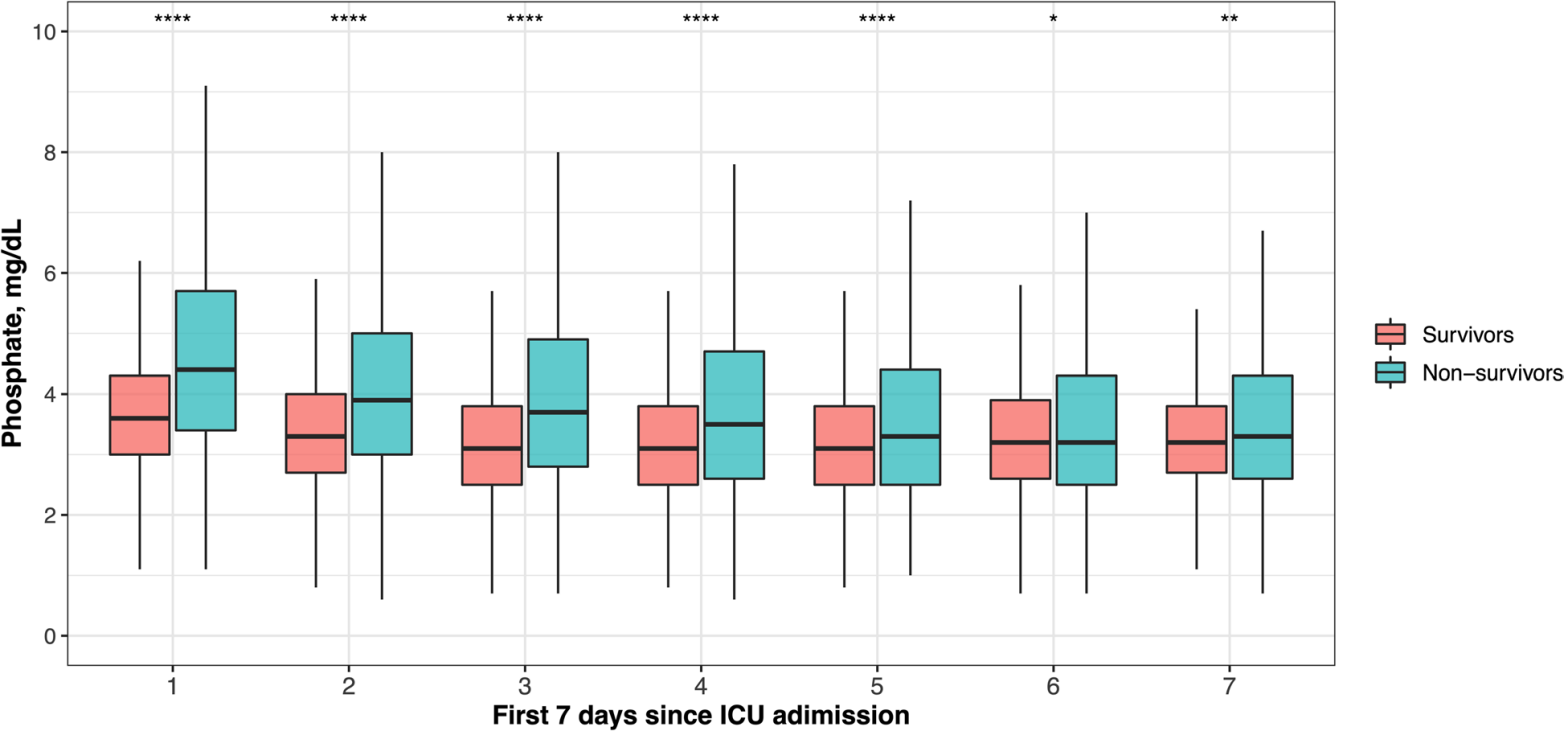
Changes of phosphate levels within the first 7 days since ICU admission between survivors and non-survivors (‘****’ 0.0001, ‘**’ 0.01, and ‘*’ 0.05). The phosphate levels of non-survivors in each day within the first 7 days were significantly higher than survivors

**Fig. 7:**
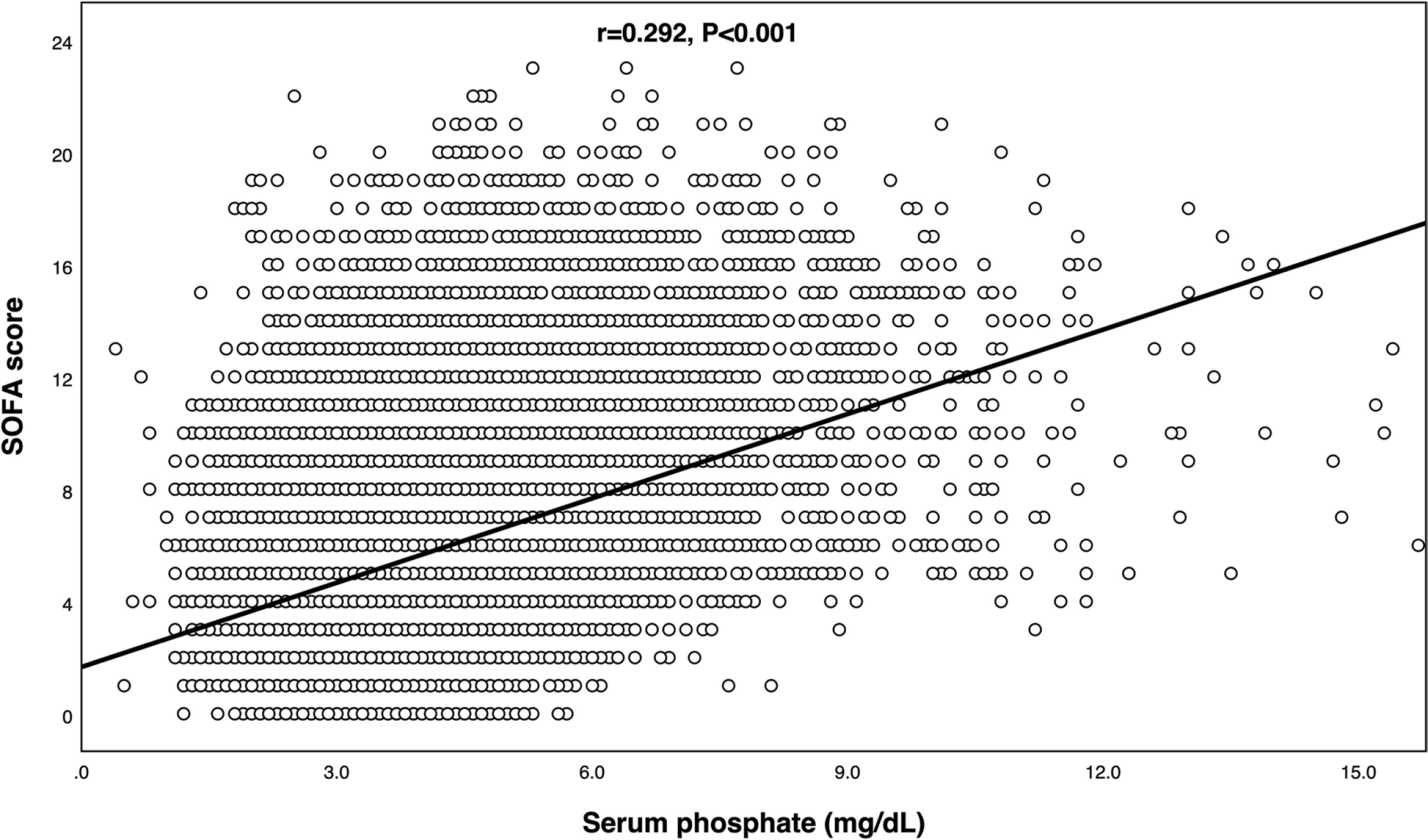
The correlation between serum phosphate and sequential organ failure assessment (SOFA) score (*r* = 0.292, *P *< 0.001)

We further conducted ROC and AUC to evaluate the predictive performance of early serum phosphate and calculate the cutoff value. Figure 3 demonstrated the results of ROC and AUC (AUC=0.67) and the cutoff value of serum phosphate was 4.3mg/dL. According to the cutoff value calculated, the survival rates between the high-phosphate group (≥4.3 mg/dL) and the low-phosphate group (<4.3 mg/dL) were compared using the log-rank test. Figure 4 demonstrated that survival probability in the low-phosphate group was significantly higher than the high-phosphate group (*P*<0.001). The restricted cubic spline model was also used to investigate the potential non-linear association between early different serum phosphate levels and risk of ICU mortality better. There was an elevated curve observed in restricted cubic spline for the association between early serum phosphate and risk of ICU mortality (Fig. 5). Figure 6 showed that phosphate levels of non-survivors in each day within the first 7 days were significantly higher than survivors. Admission serum phosphate was positively correlated with the SOFA score (Fig. 7), but the correlation was very poor.

## Discussion

The ICU mortality in critically ill elderly patients in the present study was 9.95% and this mortality was similar to other investigations reported [20, 21]. Because serum phosphate changes in hospitalized patients were common, we designed and conducted this retrospective cohort study to explore the association between early serum phosphate level and ICU mortality in critically ill elderly patients based on the large-sample database. The present study demonstrated that early elevated serum phosphate was associated with an increased risk of death in critically ill elderly patients. The result of Lowess smoothing further showed that ICU mortality increased along with serum phosphate elevating. The AUC of early serum phosphate predicting mortality was 0.67, and indicated that serum phosphate was not a good predictor in elderly critically patients and might combine with other predicters to obtain a good predictive performance. The restricted cubic spline model adjusted for related covariates was further conducted to better investigate the potential non-linear association between early different serum phosphate levels and risk of ICU mortality.

Phosphate balance is maintained due to body intake and output, and it is necessary for cell metabolism and cell function [22]. Phosphate intake is along the gastrointestinal tract through two different routes: the passive paracellular route and the active trans­cellular route [13]. When the body lacks phosphate, enteral absorption is very efficient to enable the body to maintain an optimal phosphate level. However, body balance could be disturbed due to different disease statuses. Electrolyte and mineral homeostasis disorders commonly occur in hospitalized patients. These electrolyte disorders including serum phosphate disturbances occurred more frequently in critically ill patients due to organ dysfunction [23, 24].

Haider and her (his) colleagues conducted a cross-sectional study, and found that hyperphosphatemia was usual in patients in the emergency room and associated with patient mortality [25]. Two studies conducted by Miller and HarbiIn reported that hyperphosphatemia was also associated with increased mortality in patients with mechanical ventilation or sepsis [24, 26]. In the present study, early serum phosphate in the non-survivors group was significantly higher than survivors and was independently associated with ICU mortality in critically ill elderly patients. These results indicated that early phosphate disturbance was also common in critically ill patients with elderly age and was a risk factor for poor outcomes. Therefore, physicians should pay more attention to changes of early serum phosphate in critically ill elderly patients.

The multivariate analysis also showed that the SOFA score (HR=1.122, 95%CI: 1.110-1.134) and Charlson comorbidity index (HR=1.054, 95%CI: 1.037-1.072) had a more powerful or similar association with ICU mortality, compared with serum phosphate (HR=1.056, 95%CI: 1.028-1.085). These results could be normal and accepted. The SOFA score is a sequential organ failure assessment score to describe organ dysfunction/failure including the respiratory system, cardiovascular system, coagulation system, liver, renal, and CNS [27]. It is a composite index that can reflect patient one or more organ failure and the SOFA score was more comprehensive than other indicators. Thus, the multivariate analysis in the present study showed SOFA score had a more powerful association with ICU mortality. Same as the SOFA score, the Charlson comorbidity index is also a composite index to measure comorbid disease status or casemix in health care databases [28]. In addition, the patients enrolled in the present study were elderly critically ill patients and elderly patients were usually with more comorbid diseases. Therefore, the Charlson comorbidity index might also have a more powerful or similar association with poor outcomes.

Because the present study conducted was based on a MIMIC-IV database with a large sample, we could adjust for more covariates and get more information. More than that, data in the MIMIC-IV database was collected from 2008 to 2019 and was relatively new. Thus, the result of this study was also much more credible. However, the present study also existed some limitations. Firstly, the present study could not demonstrate a causal relationship due to the design of a retrospective cohort study. Secondly, we only recorded the serum phosphate in the first day of admission and did not collect the phosphate levels at other time points. Thus, we did not have adequate evidence to conclude the possible association between the temporal change of phosphate level and patient poor outcomes. Further prospective large-sample investigations are required to solve these limitations.

## Conclusions

In critically ill elderly patients, early elevated serum phosphate was independently associated with increased ICU mortality. Therefore, when patients are on ICU admission and under treatment, clinicians should pay more attention to the change of serum phosphate in critically ill elderly patients.

## Data Availability

The datasets used for the analysis in the current study are available from the corresponding author on reasonable request.

## Abbreviations

MIMIC-IV: the medical information mart for intensive care IV
ICU: intensive care unit
SD: standard deviation
IQR: median and interquartile range
ROC: receiver operator characteristic curve
AUC: area under the curve
SOFA: sequential organ failure assessment

## References

[1] Le Borgne P, Maestraggi Q, Couraud S, Lefebvre F, Herbrecht J-E, Boivin A (2018). Critically ill elderly patients (≥ 90 years): Clinical characteristics, outcome and financial implications. PloS one.

[2] Vallet H, Moïsi L, Thomas C, Guidet B, Boumendil A (2019). Acute critically ill elderly patients: What about long term caregiver burden?. J Crit Care..

[3] Docherty AB, Anderson NH, Walsh TS, Lone NI (2016). Equity of Access to Critical Care Among Elderly Patients in Scotland: A National Cohort Study. Crit Care Med..

[4] Unsar S, Sut N (2010). Depression and health status in elderly hospitalized patients with chronic illness. Arch Gerontol Geriatr..

[5] Song X, Macknight C, Latta R, Mitnitski AB, Rockwood K (2007). Frailty and survival of rural and urban seniors: results from the Canadian Study of Health and Aging. Aging Clin Exp Res..

[6] Seckinger J, Dschietzig W, Leimenstoll G, Rob PM, Kuhlmann MK, Pommer W (2016). Morbidity, mortality and quality of life in the ageing haemodialysis population: results from the ELDERLY study. Clin Kidney J..

[7] Manning AM (2001). Electrolyte disorders. Vet Clin North Am Small Anim Pract..

[8] Umbrello M, Mantovani ES, Formenti P, Casiraghi C, Ottolina D, Taverna M, et al. Tolvaptan for hyponatremia with preserved sodium pool in critically ill patients. Annals Intensive Care. 2016;6(1). 10.1186/s13613-015-0096-2 PMC4700037 26728593

[9] Deheinzelin D, Negri EM, Tucci MR, Salem MZ, Cruz VMD, Oliveira RM (2000). Hypomagnesemia in critically ill cancer patients: a prospective study of predictive factors. Brazilian J Med Biol Res..

[10] Chen M, Sun R, Hu B (2015). The influence of serum magnesium level on the prognosis of critically ill patients. Zhonghua Wei Zhong Bing Ji Jiu yi Xue..

[11] Bhan I (2014). Phosphate management in chronic kidney disease. Curr Opin Nephrol Hypertens..

[12] Vervloet MG, van Ballegooijen AJ (2018). Prevention and treatment of hyperphosphatemia in chronic kidney disease. Kidney Int..

[13] Vervloet MG, Sezer S, Massy ZA, Johansson L, Cozzolino M, Fouque D (2017). The role of phosphate in kidney disease. Nat Rev Nephrol..

[14] Reintam Blaser A, Gunst J, Ichai C, Casaer MP, Benstoem C, Besch G (2021). Hypophosphatemia in critically ill adults and children - A systematic review. Clin Nutr..

[15] Hendrix RJ, Hastings MC, Samarin M, Hudson JQ (2020). Predictors of Hypophosphatemia and Outcomes during Continuous Renal Replacement Therapy. Blood Purif..

[16] El Shazly AN, Soliman DR, Assar EH, Behiry EG, Gad Ahmed IAEN (2017). Phosphate disturbance in critically ill children: Incidence, associated risk factors and clinical outcomes. Annals Med Surg..

[17] Thongprayoon C, Cheungpasitporn W, Mao MA, Sakhuja A, Erickson SB (2018). Admission hyperphosphatemia increases the risk of acute kidney injury in hospitalized patients. J Nephrol..

[18] Kuo G, Lee C-C, Yang S-Y, Hsiao Y-C, Chuang S-S, Chang S-W (2018). Hyperphosphatemia is associated with high mortality in severe burns. PloS One..

[19] Johnson A, Bulgarelli L, Pollard T, Horng S, Celi LA, Mark R. MIMIC-IV (version 1.0). PhysioNet. 2021. 10.13026/s6n6-xd98.

[20] Duprey MS, Van Den Boogaard M, Van Der Hoeven JG, Pickkers P, Briesacher BA, Saczynski JS, et al. Association between incident delirium and 28- and 90-day mortality in critically ill adults: a secondary analysis. Critical Care. 2020;24(1). 10.1186/s13054-020-02879-6 PMC7171767 32312288

[21] Ferrer M, Torres A (2018). Epidemiology of ICU-acquired pneumonia. Current Opinion Crit Care..

[22] Berndt T, Kumar R (2009). Novel Mechanisms in the Regulation of Phosphorus Homeostasis. Physiology.

[23] de Menezes FS, Leite HP, Fernandez J, Benzecry SG, de Carvalho WB (2006). Hypophosphatemia in children hospitalized within an intensive care unit. J Intensive Care Med..

[24] Miller CJ, Doepker BA, Springer AN, Exline MC, Phillips G, Murphy CV (2020). Impact of Serum Phosphate in Mechanically Ventilated Patients With Severe Sepsis and Septic Shock. J Intensive Care Med..

[25] Haider DG, Lindner G, Wolzt M, Ahmad SS, Sauter T, Leichtle AB (2015). Hyperphosphatemia Is an Independent Risk Factor for Mortality in Critically Ill Patients: Results from a Cross-Sectional Study. PloS One..

[26] Al Harbi SA, Al-Dorzi HM, Al Meshari AM, Tamim H, Abdukahil SAI, Sadat M, et al. Association between phosphate disturbances and mortality among critically ill patients with sepsis or septic shock. BMC. Pharmacol Toxicol. 2021;22(1). 10.1186/s40360-021-00487-w PMC8161900 34049590

[27] Vincent JL, Moreno R, Takala J, Willatts S, De Mendonca A, Bruining H (1996). The SOFA (Sepsis-related Organ Failure Assessment) score to describe organ dysfunction/failure. On behalf of the Working Group on Sepsis-Related Problems of the European Society of Intensive Care Medicine. Intensive Care Med..

[28] Sundararajan V, Henderson T, Perry C, Muggivan A, Quan H, Ghali WA (2004). New ICD-10 version of the Charlson comorbidity index predicted in-hospital mortality. J Clin Epidemiol..

